# Characteristics and Factors of 30-Day Readmissions after Hospitalization for Acute Heart Failure in China

**DOI:** 10.31083/j.rcm2508279

**Published:** 2024-08-08

**Authors:** Boxuan Pu, Wei Wang, Yanwu Yu, Yue Peng, Lubi Lei, Jingkuo Li, Lihua Zhang, Jing Li

**Affiliations:** ^1^National Clinical Research Center for Cardiovascular Diseases, NHC Key Laboratory of Clinical Research for Cardiovascular Medications, State Key Laboratory of Cardiovascular Disease, Fuwai Hospital, Chinese Academy of Medical Sciences and Peking Union Medical College, National Center for Cardiovascular Diseases, 100037 Beijing, China

**Keywords:** acute heart failure, 30-day readmission, characteristics, factors

## Abstract

**Background:**

Patients with acute heart failure (HF) are at high risk of 30-day readmission. Little is known about the characteristics and associated factors of 30-day readmissions among patients with acute HF in China.

**Methods:**

We enrolled consecutive patients hospitalized for acute HF and discharged from 52 hospitals in China from August 2016 to May 2018. We describe the rate of 30-day readmission, the time interval from discharge to readmission, and the causes of readmission. We also analyzed the factors associated with readmission risk by fitting multivariate Cox proportional hazards models.

**Results:**

We included 4875 patients with a median age of 67 years (interquartile range, 57–75), 3045 (62.5%) of whom were male. Within 30 days after discharge, 613 (12.6%) patients were readmitted for all causes, with a median from discharge to readmission of 12 (6–21) days. Most readmissions were attributed to cardiovascular causes (71.1%) and 60.0% to HF-related causes. Readmission occurred within 14 days of discharge in more than half of the patients (56.4%). Diabetes (hazard ratio [HR]: 1.25, 95% confidence interval [95% CI]: 1.06–1.50), anemia (HR: 1.26, 95% CI: 1.03–1.53), high New York Heart Association classification (HR: 1.48, 95% CI: 1.08–2.01), elevated N-terminal pro-B type natriuretic peptide (HR: 1.67, 95% CI: 1.24–2.25), and high-sensitivity cardiac troponin T (HR: 1.26, 95% CI: 1.01–1.58) were associated with increased risks of readmission. High systolic blood pressure (HR: 0.56, 95% CI: 0.38–0.81) and Kansas City Cardiomyopathy Questionnaire-12 scores (HR: 0.64, 95% CI: 0.44–0.94) were associated with decreased risk of readmission.

**Conclusions:**

In China, almost one in eight patients with acute HF were readmitted within 30 days after discharge, mainly due to cardiovascular reasons, and approximately three-fifths of the readmissions occurred in the first 14 days. Both clinical and patient-centered characteristics were associated with readmission.

## 1. Introduction

Heart failure (HF) is a major global health problem, with considerable clinical and economic burdens owing to high hospitalization and mortality rates. Concomitantly, nearly 20–25% of patients admitted with HF experienced potentially preventable hospital readmission within 30 days following discharge [1, 2, 3], which was significantly associated with high healthcare spending and adverse prognoses [4]. The Medicare Payment Advisory Commission in the U.S. reports that Medicare expenditures on potentially preventable readmissions may reach as high as $12 billion annually [2], and it was found that 30-day readmissions were related to a 2- to 3-fold greater risk of death [5]. Since readmission within 30 days post-discharge for HF has become both a recognized indicator of disease progression and the source of a considerable financial burden to the healthcare system, there is a need to research the characteristics and factors associated with 30-day readmission among patients with acute HF to improve patient outcomes and disease management.

Reducing 30-day readmissions has attracted substantial attention from policy-makers and researchers worldwide to improve the quality of care and reduce healthcare costs simultaneously. This has led present readmission monitoring programs to use the 30-day readmission rate after HF hospitalization as a reimbursement benchmark and a healthcare and hospital quality metric. Internationally, the Hospital Readmission Reduction Program in the U.S. involves public reporting of the 30-day readmission rates in hospitals and the imposed financial penalties for high readmission rates in 2012 [6]. Many European countries have also developed readmission policies to reduce 30-day readmission rates [7]. Nevertheless, national readmission reduction policies for HF among European countries and the U.S. have yet to show much success over the past two decades [8]. Moreover, in China, the Working Group on HF at the National Center for Cardiovascular Quality Improvement implemented national medical quality measures for HF, which set the 30-day HF-specific readmission rate as a quality indicator [9]. However, few studies have investigated 30-day readmission after acute HF in low- and middle-income countries (LMICs) [10]. As a populous LMIC, China has one-fifth of patients with HF worldwide, while the number of patients is expected to increase substantially [11, 12]. A few studies have suggested that approximately 10%–20% of patients with HF in China are readmitted 30 days after discharge [13, 14, 15]. However, these studies were limited by size or scope or were conducted primarily in urban settings; thus, Chinese HF patients are not well represented in the existing data. Additionally, because of regional differences in population characteristics, HF management, and healthcare system organization [16], it is necessary to investigate the 30-day readmission pattern of acute HF patients in China.

Accordingly, we used data from the China Patient-Centered Evaluative Assessment of Cardiac Events Prospective Heart Failure study (China PEACE 5p-HF study). This study included a nationwide representative sample of patients with acute HF from 52 hospitals in 20 provinces throughout mainland China between 2016 and 2018. Using these data, we aimed to (1) describe the rate and causes of 30-day readmission following acute HF hospitalization, (2) characterize the time interval between discharge and readmission within the 30-day timeframe, and (3) identify patient characteristics associated with readmission risk.

## 2. Methods

### 2.1 Study Design and Population 

The China PEACE 5p-HF study protocol has been published previously [17]. Briefly, this cohort study prospectively enrolled 4907 patients hospitalized for HF within 48 hours of admission between August 2016 and May 2018 from 52 hospitals across 20 provinces in China, with consideration of their geographical distribution and capacity to conduct the study. Eligible participants were adult local residents aged ≥18 years who were hospitalized for new-onset HF or acute decompensated chronic HF. All patients who wanted to participate provided signed written informed consent. Patients were followed up through face-to-face interviews 1, 6, and 12 months after hospital discharge. If a patient could not attend the scheduled in-person interview, study information was obtained through telephone interviews by trained staff in the national coordinating center. In this current analysis for readmission, we included all patients who were enrolled in the China PEACE 5p-HF study and discharged alive. The Ethics Committees of Fuwai Hospital and all collaborating hospitals approved the China PEACE 5p-HF study. The study was registered on https://www.clinicaltrials.gov/ (NCT02878811).

### 2.2 Data Collection 

Patient characteristics were collected via standardized questionnaires through two face-to-face interviews after patients were stable and before the discharge of the index hospitalization. Local investigators entered the data into computers equipped with a customized electronic data collection system to verify the completeness and accuracy of the entered data. The collected data were transferred to the central service, and the data quality was centrally monitored. Clinical characteristics (e.g., heart rate, systolic blood pressure (SBP), and New York Heart Association (NYHA) classification), medical history, and treatments were obtained from medical records. The record was extracted centrally by trained abstractors from electronic copies of complete medical records, using a standardized procedure and data dictionaries to ensure accuracy. Blood and urine samples were taken within 48 hours of admission for central laboratory analysis of high-sensitivity cardiac troponin T (hs-cTNT), N-terminal pro-B-type natriuretic peptide (NT-proBNP), creatinine, and glycated hemoglobin $A_{1\,c}$. Other biomarkers, such as hemoglobin and albumin, were analyzed in local laboratories.

The left ventricular ejection fraction (LVEF) was uniformly measured within 7–10 days of admission, and patients were categorized as HF with reduced ejection fraction (HFrEF, LVEF <40%), HF with mildly reduced ejection fraction (HFmrEF, 40% ≤ LVEF < 50%), or HF with preserved ejection fraction (HFpEF, LVEF ≥50%) [18]. Patients’ HF-specific health status within 48 hours of admission was evaluated by the Kansas City Cardiomyopathy Questionnaire-12 (KCCQ-12), with a summary score ranging from 0 to 100 (lower scores indicating poorer health status) [19]. Depression status was assessed by Patient Health Questionnaire-2 item (PHQ-2) [20], for which a total score ranging from 0 to 6 and ≥3 was considered to indicate depression status. Cognitive function was measured by the Mini-Cog test before discharge, with scores from 0 to 5 and ≤2 indicating cognitive impairment [21].

### 2.3 Variables Definitions

We calculated the readmission rate: The number of patients discharged and readmitted to hospitals within 30 days divided by the total number of people discharged alive. The time interval was the number of days between discharge and the first occurrence of readmission. We calculated the percentage of readmissions for the six most common reasons for readmission and the percentage of readmissions occurring each day (0–30) after discharge. Readmission for HF was defined as readmission for worsening signs or symptoms of HF resulting in the augmentation of HF therapies. Cardiovascular non-HF readmissions included readmissions related to stroke, angina, myocardial infarction, and other cardiovascular diseases. Readmissions without HF or cardiovascular non-HF reasons were defined as non-cardiovascular readmissions. Additionally, we examined those reasons for readmission during cumulative time periods after discharge (0–3, 0–7, 0–15, and 0–30 days) and during consecutive time periods (0–3, 4–7, 8–15, and 16–30 days) to provide information about the diversity and variation of readmission causes within 30 days of discharge. When investigating factors associated with readmission risk, NT-proBNP was classified into four quartiles, and the diagnostic criteria of the laboratory classified the biomarkers, including hs-cTNT, sodium, potassium, creatine, and albumin.

### 2.4 Outcomes

The outcome of this study was the first unplanned readmission within 30 days of discharge from the index hospitalization for acute HF. Unplanned readmission was defined as any new index hospitalization excluding the index hospitalization claim, transfers from another hospital, admissions for rehabilitation, or elective or unknown admissions. Each readmission record was determined according to the interview and medical record of each patient. Outcome events were centrally adjudicated by trained clinicians.

### 2.5 Statistical Analyses

We reported median (interquartile range (IQR)) and counts (percentages) for categorical and continuous variables. We used the Kruskal–Wallis test to compare continuous variables and Pearson’s chi-square test for categorical variables for the distribution of the characteristics between patients with and without 30-day readmission.

We fitted multivariate Cox proportional hazards models to analyze the association between 30-day all-cause readmission and patient characteristics. All admission characteristics listed in Table 1 were included in the multivariable model as candidate variables. In a sensitivity analysis, we fitted a multivariate Cox model using the shared frailty approach with a gamma distribution, considering hospitals as random effects and censoring for death to control the competing risks of death. There were missing data on covariates ranging from 0.02% to 6.22% (**Supplementary Table 1**). Multiple Markov chain Monte Carlo method imputations were used to impute the missing data. A two-sided *p*-value of <0.05 was considered to indicate statistical significance. All analyses were performed with SAS version 9.4 (SAS Institute Inc, Cary, NC, USA).

**Table 1.** **Characteristics of the study participants**.

Characteristics			Total (n = 4875)	Readmitted within 30 d (n = 613)	Not readmitted within 30 d (n = 4262)	*p*-value
Demographics						
	Age, years, median (IQR)		67 (57–75)	67 (58–76)	67 (57–75)	0.151
	Male, n (%)		3045 (62.5)	376 (61.3)	2669 (62.6)	0.539
Heart failure history, n (%)						
	DCHF		3415 (70.1)	452 (73.7)	2963 (69.5)	0.033
Medical history, n (%)						
	Hypertension		2845 (58.4)	357 (58.2)	2488 (58.4)	0.948
	Coronary heart disease		2818 (57.8)	374 (61.0)	2444 (57.3)	0.086
	Atrial fibrillation		1777 (36.5)	242 (39.5)	1535 (36.0)	0.096
	Valvular heart disease		794 (16.3)	120 (19.6)	674 (15.8)	0.018
	Diabetes mellitus		1542 (31.6)	233 (38.0)	1309 (30.7)	<0.001
	Anemia		897 (18.4)	155 (25.3)	742 (17.4)	<0.001
	COPD		950 (19.5)	116 (18.9)	834 (19.6)	0.706
	Stroke		999 (20.5)	119 (19.4)	880 (20.6)	0.479
	Renal dysfunction		1398 (28.7)	218 (35.6)	1180 (27.7)	<0.001
Clinical features						
	SBP, mmHg, median (IQR)		130 (116–148)	125 (110–140)	130 (118–150)	<0.001
	DBP, mmHg, median (IQR)		80 (70–90)	78 (70–87)	80 (70–90)	<0.001
	NYHA class. n (%)					<0.001
		II	704 (14.4)	55 (9.0)	649 (15.2)	
		III	2160 (44.3)	259 (42.2)	1901 (44.6)	
		IV	2011 (41.3)	299 (48.8)	1712 (40.2)	
	LVEF, %, median (IQR)		43 (33–56)	43 (32–55)	44 (33–57)	0.114
	LVEF subtypes, n (%)					0.504
		HFrEF	1848 (37.9)	237 (38.7)	1611 (37.8)	
		HFmrEF	1329 (27.3)	175 (28.5)	1154 (27.1)	
		HFpEF	1698 (34.8)	201 (32.8)	1497 (35.1)	
Laboratory tests						
	hs-cTNT, ng/L, median (IQR)		21 (13–40)	28 (16–51)	21 (12–39)	<0.001
	NT-proBNP, pg/mL, median (IQR)		1486 (609–3311)	2147 (933–4693)	1427 (565–3169)	<0.001
	Serum creatinine, µmol/L, median (IQR)		92 (77–111)	96 (79–119)	91 (76–110)	<0.001
	Serum sodium, mmol/L, median (IQR)		140 (137–142)	139 (136–142)	140 (137–142)	<0.001
	Serum potassium, mmol/L, median (IQR)		4.1 (3.7–4.4)	4.1 (3.7–4.5)	4.1 (3.7–4.4)	0.041
	Albumin, g/L, median (IQR)		39 (36–42)	38 (35–41)	39 (36–42)	<0.001
Health status						
	KCCQ-12 score, median (IQR)		43 (27–61)	38 (22–57)	44 (28–61)	<0.001
Depression						
	PHQ-2 score, median (IQR)		4 (2–5)	4 (2–6)	4 (2–5)	<0.001
	Depression status, n (%)		2994 (61.4)	416 (67.9)	2578 (60.5)	<0.001
Cognitive function						
	Mini-Cog score, median (IQR)		4 (2–5)	3 (2–5)	4 (2–5)	0.064
	Cognitive impairment, n (%)		1762 (36.1)	238 (38.8)	1524 (35.8)	0.139
	Length of stay, day, median (IQR)		9 (7–13)	10 (7–14)	9 (7–13)	<0.001
Treatment at discharge, n (%)						
	ACEI or ARB		2541 (52.1)	278 (45.4)	2263 (53.1)	<0.001
	β-blockers		2879 (59.1)	344 (56.1)	2535 (59.5)	0.114
	Aldosterone antagonists		3100 (63.6)	375 (61.2)	2725 (63.9)	0.184

Abbreviations: IQR, interquartile range; DCHF, decompensated chronic heart failure; SBP, systolic blood pressure; DBP, diastolic blood pressure; NYHA class, New York Heart Association classification; COPD, chronic obstructive pulmonary disease; LVEF, left ventricular ejection fraction; HFrEF, heart failure with reduced ejection fraction; HFmrEF, heart failure with mildly reduced ejection fraction; HFpEF, heart failure with preserved ejection fraction; hs-cTNT, high-sensitivity cardiac troponin T; NT-proBNP, N-terminal pro-B type natriuretic peptide; KCCQ-12, Kansas City Cardiomyopathy Questionnaire-12; PHQ-2, Patient Health Questionnaire-2 item; ACEI, angiotensin-converting enzyme inhibitor; ARB, angiotensin receptor blocker.

## 3. Results

### 3.1 Characteristics of the Study Participants 

Among the 4907 patients, we excluded 32 who died during the index hospitalization and included 4875 patients in this current analysis. The median age was 67 years (IQR: 57–75), 62.5% were men, and 70.1% had decompensated chronic heart failure (DCHF). Comorbidities such as hypertension (58.4%), coronary heart disease (57.8%), and atrial fibrillation (36.5%) were common. The median KCCQ-12 score was 43 (IQR: 27–61), and conditions such as depression status (61.4%) and cognitive impairment (36.1%) were prevalent. The median length of stay (LOS) was 9 days (IQR: 7–13). Table 1 shows the admission characteristics of patients with or without 30-day readmission.

Within 30 days following discharge, 613 patients (12.6%) were readmitted for any cause. Compared to patients without 30-day readmission, those with readmission were more likely to have DCHF; have comorbidities, depression status, worse health status; have a higher NYHA classification, hs-cTNT, and NT-proBNP; have longer LOS; and be less frequently prescribed angiotensin-converting enzyme inhibitor/angiotensin receptor blockers (angiotensin-converting enzyme inhibitor (ACEI)/angiotensin receptor blocker (ARBs)) at discharge.

### 3.2 Causes of 30-Day Readmission 

**Fig. 1.**
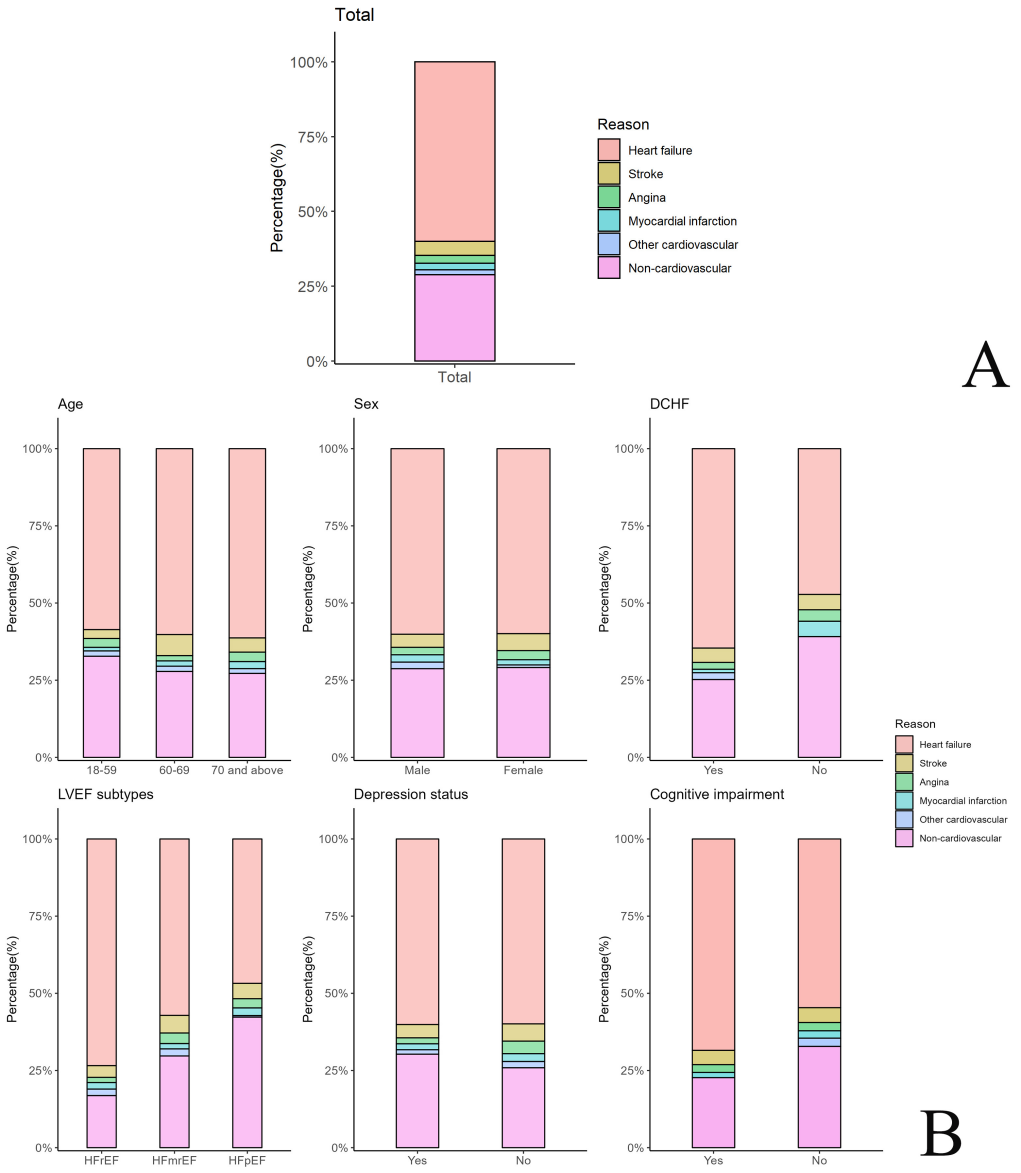
**Distribution of causes for readmission within 30 days following discharge among patients with acute heart failure, and among demographic and clinical characteristic subgroups**. (A) Distribution of causes for 30-day readmission among all study participants. (B) Distribution of causes for 30-day readmission in subgroups by selected demographics (age, sex, DCHF) and clinical characteristics (LVEF subtypes, depression status, cognitive impairment). Abbreviations: DCHF, decompensated chronic heart failure; LVEF, left ventricular ejection fraction; HFrEF, heart failure with reduced ejection fraction; HFmrEF, heart failure with mildly reduced ejection fraction; HFpEF, heart failure with preserved ejection fraction.

A total of 436 patients (71.1%) were readmitted for cardiovascular causes within 30 days following discharge. Among those, there were 368 (60.0%) readmissions due to HF, 29 (4.7%) due to stroke, 16 (2.6%) due to angina, 13 (2.1%) due to myocardial infarction, and 10 (1.6%) due to other cardiovascular events, such as atrial fibrillation, implantable cardio vision defibrillator, or cardiac resynchronization therapy (Fig. 1). Overall, HF was the dominant single cause of readmission, and 245 patients (40.0%) were readmitted for non-HF-related causes. A total of 177 patients (28.9%) were readmitted for non-cardiovascular causes, including respiratory disease, cancer, and infectious diseases. The distribution of causes for readmission stratified by patient characteristics, including sex, age, DCHF, LVEF subtypes, depression status, and cognitive impairment, is displayed in** Supplementary Table 2**. 

### 3.3 Time Interval between Discharge and Readmission

**Fig. 2.**
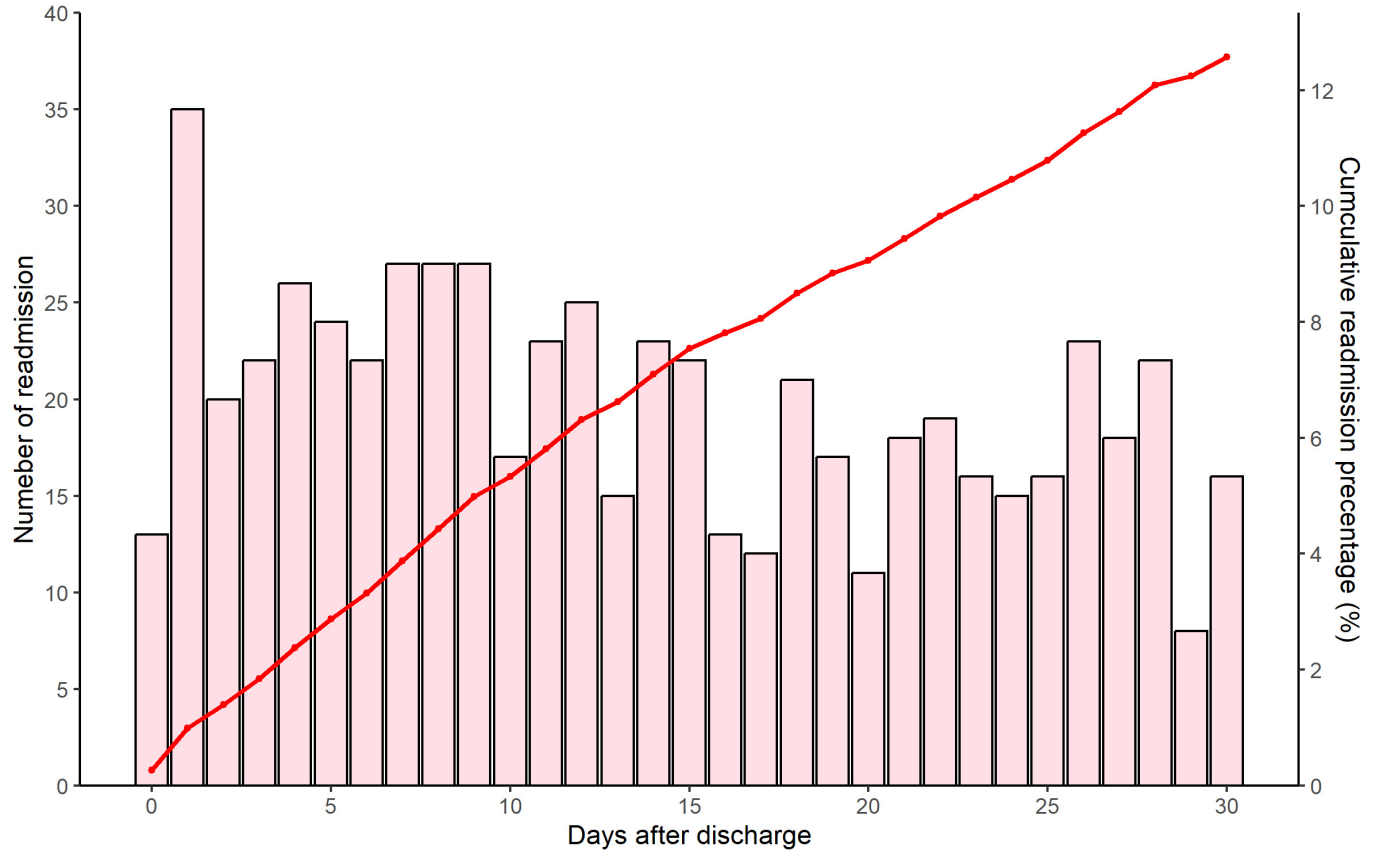
**Distribution of time intervals between discharge and readmission within 30 days following discharge among patients with acute heart failure**. Note: days after discharge is the time interval between discharge and readmission.

**Fig. 3.**
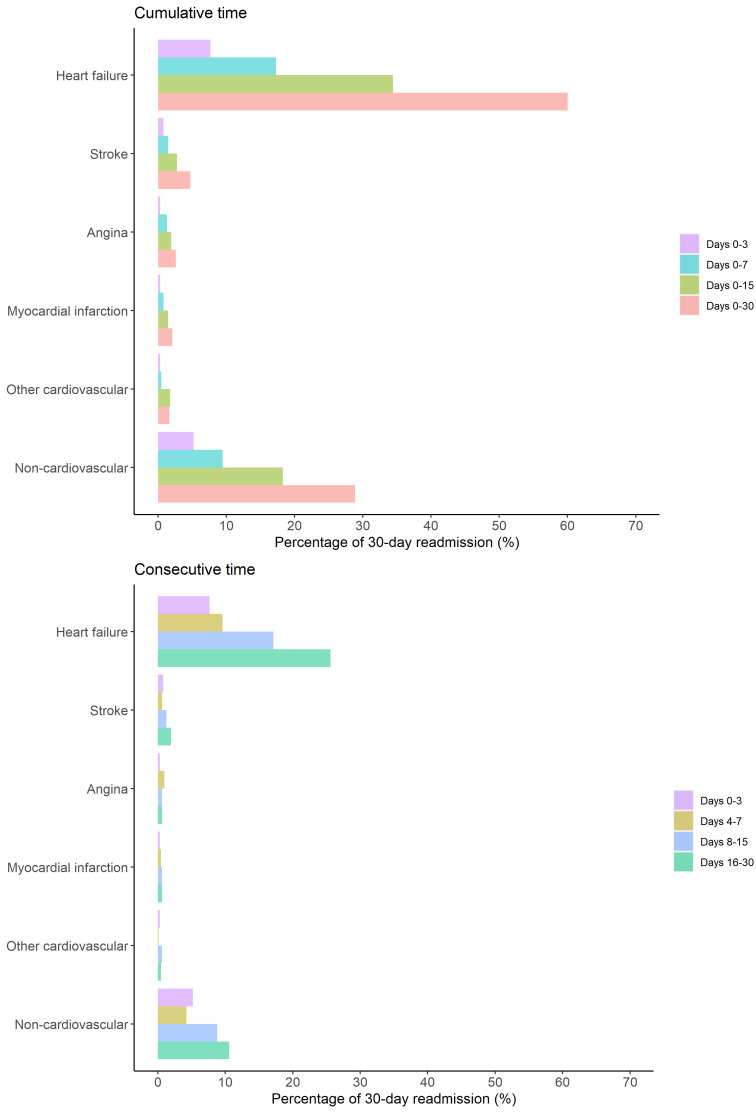
**Distribution of readmission causes according to cumulative time and consecutive periods within 30 days following discharge among patients with acute heart failure**.

The median time interval of readmission was 12 days (IQR: 6–21) from the date of discharge to readmission (Fig. 2, **Supplementary Fig. 1**). A total of 14.7%, 30.8%, and 60.0% of all 30-day readmissions occurred during days 0–3, 0–7, and 0–15, respectively, following discharge, while 40% of the readmissions occurred within 16–30 days after discharge. When considering the change in readmission causes over time (Fig. 3, **Supplementary Fig. 2**), the pattern of causes for readmission was similar for cumulative and consecutive periods after discharge. The percentages of HF-related readmissions were 7.7%, 17.3%, 34.4%, and 60.0% at 0–3, 0–7, 0–15, and 0–30 days after discharge, respectively. The median time intervals of readmission were 14 days (IQR: 7–22) and 12 days (IQR: 6–20) for HF-related and non-HF-related causes, respectively (**Supplementary Fig. 3**).

### 3.4 Associated Factors of 30-Day Readmission

Multivariate analysis revealed the following factors to be associated with higher risks of 30-day readmission: diabetes mellitus (hazard ratio (HR): 1.25, 95% confidence interval (CI): 1.06–1.50, *p* = 0.010), anemia (HR: 1.26, 95% CI: 1.03–1.53, *p* = 0.026), high NYHA classification (Ⅳ: HR: 1.48, 95% CI: 1.08–2.01, *p* = 0.014), elevated NT-proBNP (Q2 [617–1521 pg/mL]: HR: 1.67, 95% CI: 1.27–2.20, *p* < 0.01; Q3 [1522–3438 pg/mL]: HR: 1.46, 95% CI: 1.10–1.94, *p* = 0.010; Q4 [≥3439 pg/mL]: HR: 1.67, 95% CI: 1.24–2.25, *p* < 0.01), and high hs-cTNT (>14 ng/L: HR 1.26, 95% CI: 1.01–1.58, *p* = 0.041).

**Fig. 4.**
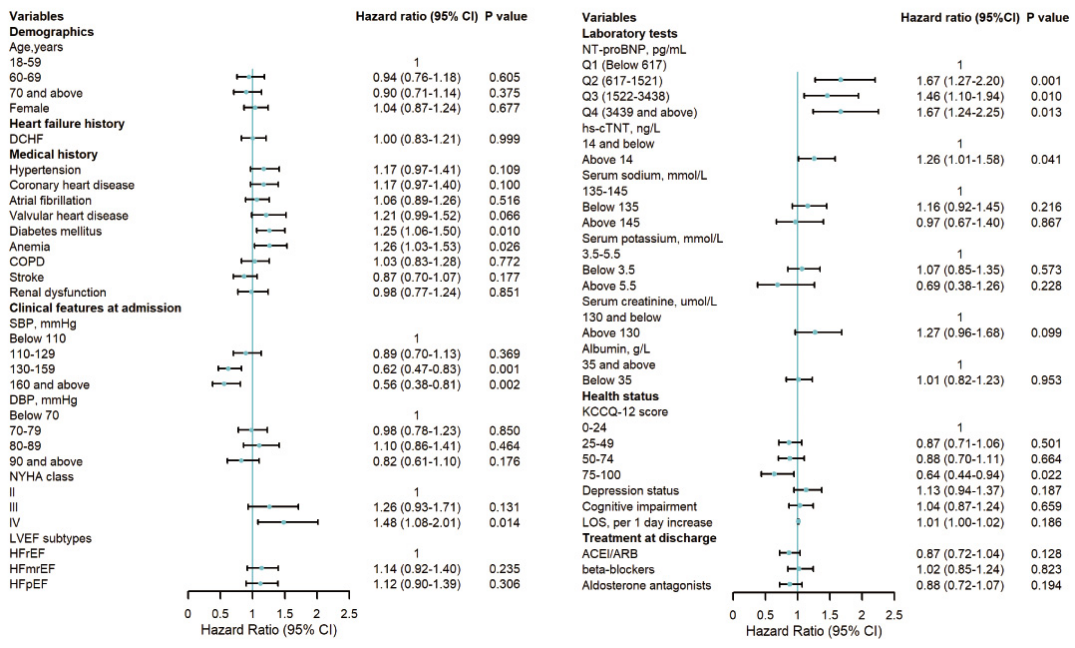
**Associated factors of 30-day readmission among patients with acute heart failure**. Abbreviations: CI, confidence interval; DCHF, decompensated chronic heart failure; SBP, systolic blood pressure; DBP, diastolic blood pressure; NYHA class, New York Heart Association classification; COPD, chronic obstructive pulmonary disease; LVEF, left ventricular ejection fraction; HFrEF, heart failure with reduced ejection fraction; HFmrEF, heart failure with mildly reduced ejection fraction; HFpEF, heart failure with preserved ejection fraction; hs-cTNT, high-sensitivity cardiac troponin T; NT-proBNP, N-terminal pro-B type natriuretic peptide; KCCQ-12, Kansas City Cardiomyopathy Questionnaire-12; LOS, length of stay; ACEI, angiotensin-converting enzyme inhibitor; ARB, angiotensin receptor blocker.

In contrast, SBP ≥130 mmHg (130–159 mmHg: HR: 0.62, 95% CI: 0.47–0.83, *p* < 0.01; ≥160 mmHg: HR: 0.56, 95% CI: 0.38–0.81, *p* < 0.01) and KCCQ-12 scores of 75–100 (HR: 0.64, 95% CI: 0.44–0.94, *p* = 0.022) were associated with decreased risk of readmission (Fig. 4). There were 55 (1.1%) deaths that occurred within 30 days after discharge. The results were approximately similar in a sensitivity analysis considering the competing risks of death (**Supplementary Fig. 4**).

## 4. Discussion

Using a nationwide, multicenter, prospective cohort of hospitalized patients with acute HF, this study is the first to investigate the rate, causes, time interval, and associated factors of 30-day readmission for patients with acute HF in China. Within 30 days after discharge, approximately one-eighth of patients with acute HF were readmitted, with a median time of 12 days from the date of discharge to readmission. Most readmissions were attributed to HF-related or non-cardiovascular causes, with three-fifths occurring 14 days after discharge. SBP ≤130 mmHg, higher level of NYHA classification, NT-proBNP, hs-cTNT, diabetes mellitus, anemia, and worse health status in index hospitalization were associated factors with increased risks of 30-day readmission.

We first reported the rate of 30-day readmission in Chinese patients with acute HF, a large population that has yet to be well represented in prior data. Our study observed that the 30-day readmission rate was nearly 12.6% in patients with acute HF, which was lower than that in other large prospective cohorts in Europe and the United States, where approximately 25% of patients were readmitted within 30 days [22, 23, 24]. Furthermore, a meta-analysis of data from 38 countries reported a pooled 30-day readmission rate of 13.2%. (10.5%–16.1%) and there was substantial variability in readmission rates both globally and within continents [25]. Compared with other prospective studies of acute HF, especially in Western countries, our study showed some substantial disparities in the patients’ characteristics, such as a higher proportion of young patients, a longer LOS, a lower proportion of patients with HFrEF, and low comorbid burdens, such as hypertension and diabetes mellitus (**Supplementary Table 3**).

Regarding the causes of readmission, most readmissions occurred due to cardiovascular causes, and HF was the most common single cause, accounting for more than half of the readmissions. Incomplete decongestion during hospitalization or rapid recurrence of congestion in the early post-discharge interval may also cause recurrent HF [26]. A prospective cohort in Spain and data for Medicare patients in the U.S. also found that HF was the most common specific cause of readmission [27, 28, 29]. However, previous studies using data from the two clinical trials in patients with acute HF yielded different results; most readmissions were attributed to reasons other than HF [30, 31]. Differences in study designs and age groups could explain this discrepancy. Moreover, non-cardiovascular causes of readmission were also common in readmitted patients, which suggests that the diversity of causes of readmission reflects the increased vulnerability of patients with acute HF and the need for multidisciplinary teams to reduce readmission.

Similar to the previous studies of the Medicare population [28], the Italian nationwide cardiology registry [32], and randomized HF trials in the United States [33], we found that patients were readmitted more frequently in the first 1–2 weeks of the 30 days following discharge. The reasons underlying these findings may be associated with factors of the index hospitalization, including acute illness severity and inpatient care processes [34]. These findings highlight the importance of transitional care and early physician follow-up appointments within 1–2 weeks after discharge for decreased readmission [18, 35]. Furthermore, we found no substantial change in the overall pattern of causes for readmission within 30 days of discharge, which suggests that outpatient physicians should know that diverse causes of readmission are generally stable over time following discharge and should implement sustainable monitoring and preventive measures.

Several patient-related factors during the index hospitalization could predict readmission after acute HF, suggesting that the risk of 30-day readmission could be predicted before discharge. We identified certain clinical characteristics and laboratory biomarkers related to increased risks of 30-day readmission, such as diabetes mellitus, anemia, high NYHA classification, NT-proBNP, and hs-cTNT. These characteristics are similar to those in other studies that evaluated 30-day readmission [30]. In addition to those clinical factors, patient-centered variables, such as worse HF-specific health status, which are rarely measured in daily care for acute HF, were associated with increased readmission risk. Thus, our findings reinforce the growing importance of patient-centered variables. In contrast, patients with an SBP ≥130 mmHg were at a lower risk of readmission, which might suggest a nonlinear relationship between SBP and the risk of readmission. Measuring these clinical and patient-centered variables during hospitalization could help physicians facilitate the identification of acute HF patients at increased risk of readmission and improve the promptness of follow-up healthcare.

Our study has several important implications. At the individual level, our findings could help physicians identify patients at high risk of 30-day readmission during index hospitalization and pay attention to multidisciplinary transitions of care and readmission prevention strategies earlier after discharge. Additionally, contemporary HF guidelines and recommendations emphasize the importance of reducing 30-day readmission and focusing on HF-specific strategies. Thus, from a health-policy perspective, the present analysis complements prior studies in China, guides health policy agencies to recognize the burden and pattern of 30-day readmission for patients with acute HF, and reinforces the need for specific healthcare policies and strategies to reduce 30-day readmissions in China.

The strength of this study was the large number of hospitalizations for acute HF included in a nationwide, real-world, prospective cohort in China. To our knowledge, our study is the first to evaluate the rate, time interval, causes, and associated factors of readmission in a nationally representative sample of Chinese patients with acute HF. However, several limitations should be acknowledged. First, the 30-day time limit is an artificial endpoint, although it is currently used as a quality metric in health systems. Second, given the observational nature of the analysis, residual confounding effects, such as nutrition and environmental factors, could not be ruled out entirely. Finally, changes in medical treatment and prevention strategies after discharge over time were not assessed as such data are unavailable.

## 5. Conclusions

In this national prospective cohort study in China, nearly one in eight patients hospitalized for acute HF were readmitted within 30 days of discharge, mainly for cardiovascular reasons such as HF, and approximately three-fifths of the readmissions occurred in the first 14 days. Moreover, the risk of readmission was associated with clinical and patient-centered characteristics. This study provides nationally representative data on the characteristics and associated factors of 30-day readmissions for patients with acute HF in China, which will help physicians identify patients at high risk for readmission and target preventive care policies to reduce 30-day readmission rates.

## Data Availability

The datasets generated and/or analyzed during the current study are not publicly available due to the government policy stipulates, it is not permissible for the researchers to make the raw data publicly available at this time. And currently, it is not yet possible for other researchers to apply for the access.

## Supplementary Material

Supplementary material associated with this article can be found, in the online version, at https://doi.org/10.31083/j.rcm2508279.
