# Supplementary material for: Characteristics and Factors of 30-Day Readmissions after Hospitalization for Acute Heart Failure in China
Source: Rev Cardiovasc Med. 2024 Aug 8;25(8):279. doi: 10.31083/j.rcm2508279 (PMC11366993; doi:10.31083/j.rcm2508279)
Supplement: Supplementary file 1 [file 2153-8174-25-8-279-s1.docx]

**Supplementary material**

Tables of contents

Supplementary Table 1. Proportions of missing data in covariates

Supplementary Table 2. Characteristics of study participants by causes of readmission

Supplementary Table 3. Comparison of China PEACE 5p-HF study with other prospective studies of acute heart failure

Supplementary Fig. 1. Density distribution of readmission within 30 days following discharge among patients with acute heart failure

Supplementary Fig. 2. Distribution of readmission causes by time interval of readmission within 30 days following discharge among patients with acute heart failure

Supplementary Fig. 3. Distribution of time interval for heart failure (HF) readmission and non-HF readmission within 30 days following discharge among patients with acute heart failure

Supplementary Fig. 4. Associated factors of 30-day readmission following discharge among patients with acute heart failure considering the competing risk

**Supplementary Table 1.** **Proportions of missing data in covariates**

| Covariates | N | Missing (n, %) |
| --- | --- | --- |
| SBP | 4874 | 1 (0.02) |
| DBP | 4874 | 1 (0.02) |
| NT-proBNP | 4764 | 111 (2.28) |
| Creatinine | 4869 | 6 (0.12) |
| Sodium | 4807 | 68 (1.39) |
| Potassium | 4807 | 68 (1.39) |
| Albumin | 4651 | 224 (4.59) |
| LVEF | 4582 | 293 (6.01) |
| hs-cTNT | 4718 | 157 (3.22) |
| KCCQ-12 score | 4839 | 36 (0.74) |
| PHQ-2 score | 4572 | 303 (6.22) |
| Mini-cog score | 4572 | 303 (6.22) |
| Abbreviations: SBP: systolic blood pressure; DBP: diastolic blood pressure; NT-proBNP: N-terminal pro-B type natriuretic peptide; LVEF: left ventricular ejection fraction; hs-cTNT: high-sensitivity cardiac troponin T; KCCQ-12: Kansas City Cardiomyopathy Questionnaire-12; PHQ-2: Patient Health Questionnaire-2 item. | | |

**Supplementary Table 2. Characteristics of study participants by causes of readmission**

| **Variables** | **Total** | **Heart failure** | **Stroke** | **Angina** | **Myocardial**  **infarction** | **Other**  **cardiovascular** | **Non-cardiovascular** |
| --- | --- | --- | --- | --- | --- | --- | --- |
| Total, n | 613 | 368 | 29 | 16 | 13 | 10 | 177 |
| Sex, n (%) |  |  |  |  |  |  |  |
| Male | 376 (61.3) | 226 (61.4) | 16 (55.2) | 9 (56.2) | 9 (69.2) | 8 (80.0) | 108 (61.0) |
| Female | 237 (38.7) | 142 (38.6) | 13 (44.8) | 7 (43.8) | 4 (30.8) | 2 (20.0) | 69 (39.0) |
| Age, years, n (%) |  |  |  |  |  |  |  |
| 18-59 | 174 (28.4) | 102 (27.7) | 5 (17.2) | 5 (31.2) | 2 (15.4) | 3 (30.0) | 57 (32.2) |
| 60-69 | 176 (28.7) | 106 (28.8) | 12 (41.4) | 3 (18.8) | 3 (23.1) | 3 (30.0) | 49 (27.7) |
| 70 and above | 263 (42.9) | 160 (43.5) | 12 (41.4) | 8 (50.0) | 8 (61.5) | 4 (40.0) | 71 (40.1) |
| DCHF, n (%) |  |  |  |  |  |  |  |
| Yes | 452 (73.7) | 292 (79.3) | 21 (72.4) | 10 (62.5) | 5 (38.5) | 10 (100.0) | 114 (64.4) |
| No | 161 (26.3) | 76 (20.7) | 8 (27.6) | 6 (37.5) | 8 (61.5) | 0 | 63 (35.6) |
| LVEF subtypes, n (%) |  |  |  |  |  |  |  |
| HFrEF | 237 (38.7) | 174 (47.3) | 9 (31.0) | 4 (25.0) | 5 (38.5) | 5 (50.0) | 40 (22.6) |
| HFmrEF | 175 (28.5) | 100 (27.2) | 10 (34.5) | 6 (37.5) | 3 (23.1) | 4 (40.0) | 52 (29.4) |
| HFpEF | 201 (32.8) | 94 (25.5) | 10 (34.5) | 6 (37.5) | 5 (38.5) | 1 (10.0) | 85 (48.0) |
| Depression status, n (%) |  |  |  |  |  |  |  |
| Yes | 416 (67.9) | 250 (67.9) | 18 (62.1) | 8 (50.0) | 8 (61.5) | 6 (60.0) | 126 (71.2) |
| No | 197 (32.1) | 118 (32.1) | 11 (37.9) | 8 (50.0) | 5 (38.5) | 4 (40.0) | 51 (28.8) |
| Cognitive impairment, n (%) |  |  |  |  |  |  |  |
| Yes | 238 (38.8) | 163 (44.3) | 11 (37.9) | 6 (37.5) | 4 (30.8) | 0 | 54 (30.5) |
| No | 375 (61.2) | 205 (55.7) | 18 (62.1) | 10 (62.5) | 9 (69.2) | 10 (100.0) | 123 (69.5) |

Abbreviations: DCHF: decompensated chronic heart failure; LVEF: left ventricular ejection fraction; HFrEF: heart failure with reduced ejection fraction; HFmrEF: heart failure with mildly reduced ejection fraction; HFpEF; heart failure with preserved ejection fraction.

**Supplementary Table 3. Comparison of China PEACE 5p-HF study with other prospective studies of acute heart failure**

|  | **China PEACE 5p-HF** | **IN-HF**  **Outcome Registry^1^** | **NRD^2^** | **KorAHF^3^** | **ICCNHFR^4^** | **ATTEND^5^** | **GWTG-HF^6^** |
| --- | --- | --- | --- | --- | --- | --- | --- |
| **Time frame** | 2016-2018 | 2007-2009 | 2013-2014 | 2011-2014 | 2018-2019 | 2007-2011 | 2005-2011 |
| **Regions/countries** | China | Italian | United States | Korea | India | Japan | United States |
| **Patients number** | 4875 | 1520 | 546841 | 5625 | 5269 | 4842 | 56477 |
| **Age, year (mean ± SD, median/IQR)** | 67 (57-75) | 72±11 | 75 (63-85) | 68.5±14.5 | 61.9±13.9 | 73.0±14.0 | 80 (74-86) |
| **Female (%)** | 37.5 | 40.0 | 49.2 | 46.8 | 32.9 | 42.0 | 54.5 |
| **History of heart failure (%)** | 70.5 | 58.0 | N/A | 47.8 | 66.4 | 36.0 | 53.7 |
| **Medical history** (%) |  |  |  |  |  |  |  |
| Hypertension | 58.4 | 64.0 | 77.5 | 62.2 | 52.3 | 69.0 | 75.7 |
| CAD | 57.8 | N/A | N/A | 42.9 | N/A | N/A | 51.7 |
| Diabetes mellitus | 31.6 | 41.0 | 44.5 | 40.0 | 51.5 | 34.0 | 39.2 |
| Renal dysfunction | 28.7 | 31.0 | 41.6 | 14.3 | 14.8 | N/A | 19.2 |
| Atrial fibrillation | 36.5 | 39.0 | 41.8 | 28.5 | N/A | 36.0 | 38.6 |
| COPD | 19.5 | 31.0 | 36.6 | 11.3 | 9.4 | 10.0 | 29.3 |
| Anemia | 18.4 | N/A | 29.6 | N/A | N/A | N/A | 18.8 |
| VHD | 16.3 | 20 | N/A | N/A | N/A | N/A | 13.8 |
| Stroke | 20.5 | N/A | N/A | 15.2 | 5.0 | 14.0 | 16.7 |
| PAD | 12.4 | 20.0 | 12.6 | N/A | 2.5 | N/A | 13.5 |
| **Echocardiogram** |  |  |  |  |  |  |  |
| LVEF (%), median (IQR) | 44 (33-56) | 38±14 | N/A | 37.7±15.6 | N/A | 45 (30-57) | 45 (30-57) |
| LVEF<40% (%) | 37.9 | 57.0 | N/A | 60.5 | 68.3 | 53.0 | 39.3 |
| LVEF 40-49 (%) | 27.3 | 19.0 | N/A | 14.3 | N/A | N/A | 13.6 |
| LVEF≥50% (%) | 34.8 | 24.0 | N/A | 25.2 | N/A | N/A | 47.1 |
| **Length of stay, median (IQR)** | 9 (7-13) | 12±10 | 4 (2-6) | 9 (1-311) | 6±5 | 21(14-32) | 4 (3-7) |
| **Discharge medication (%)** |  |  |  |  |  |  |  |
| ACEI/ARB | 52.1 | 76.0 | N/A | 65.9 | 41.2 | N/A | 61.8 |
| Beta-blocker | 59.0 | 61.0 | N/A | 49.9 | 67.2 | N/A | 77.2 |
| Diuretic | 68.9 | 25.0 | N/A | N/A | 88.0 | N/A | 47.8 |
| Aldosterone antagonist | 63.6 | 54.0 | N/A | 44.9 | N/A | N/A | 13.2 |
| **30-day readmission (%)** |  |  |  |  |  |  |  |
| All-cause readmission | 12.6 | 6.2 | 20.2 | N/A | 8.0 | N/A | 21.2 |
| HF-related readmission | 7.6 | 5.6 | 6.7 | 7.0 | N/A | 4.6 | N/A |

Abbreviations: SD, standard deviation; HF: heart failure; CAD: coronary heart disease; COPD: chronic obstructive pulmonary disease; VHD: valvular heart disease; PAD: peripheral arterial disease; LVEF: left ventricular ejection fraction; ACEI: angiotensin-converting enzyme inhibitors; ARB: angiotensin receptor blocker; CV: cardiovascular. China PEACE 5p-HF: China Patient-centered Evaluative Assessment of Cardiac Events Prospective Heart Failure Study; IN-HF Outcome Registry: Italian-heart failure outcome registry; NRD: Ntional Readmission Database; KorAHF: the Korean Acute Heart Failure Registry; ICCNHFR: Indian College of Cardiology National Heart Failure Registry; ATTEND: Acute decompensated heart failure syndrome registry; GWTG-HF: the American Heart Association Get With the Guidelines Heart Failure registry.

1. Di Tano G, De Maria R, Gonzini L, Aspromonte N, Di Lenarda A, et al; The 30-day metric in acute heart failure revisited: data from IN-HF Outcome, an Italian nationwide cardiology registry. Eur J Heart Fail. 2015;17(10):1032-41. doi: 10.1002/ejhf.290.

2. Patil S, Shah M, Patel B, Agarwal M, Ram P, et al. Readmissions among patients admitted with acute decompensated heart failure based on income quartiles. Mayo Clin Proc. 2019; 94(10):1939-1950. doi: 10.1016/j.mayocp.2019.05.027. =

3. Lee SE, Lee HY, Cho HJ, Choe WS, Kim H, et al. Clinical characteristics and outcome of acute heart failure in Korea: results from the Korean Acute Heart Failure Registry (KorAHF). Korean Circ J. 2017;47(3):341-353. doi: 10.4070/kcj.2016.0419.

4. Jayagopal PB, Sastry SL, Nanjappa V, Abdullakutty J, Joseph J, et al. Clinical characteristics and 30-day outcomes in patients with acute decompensated heart failure: results from Indian College of Cardiology National Heart Failure Registry (ICCNHFR). Int J Cardiol. 2022; 356:73-78. doi: 10.1016/j.ijcard.2022.03.021.

5. Shiraishi Y, Kohsaka S, Sato N, Takano T, Kitai T, et al. 9-Year trend in the management of acute heart failure in Japan: a report from the National Consortium of Acute Heart Failure Registries. J Am Heart Assoc. 2018;7(18): e008687. doi: 10.1161/JAHA.118.008687.

6. Frizzell JD, Liang L, Schulte PJ, Yancy CW, Heidenreich PA, et al. Prediction of 30-day all-cause readmissions in patients hospitalized for heart failure: comparison of machine learning and other statistical approaches. JAMA Cardiol. 2017;2(2):204-209. doi: 10.1001/jamacardio.2016.3956.

**Supplementary Fig. 1.**

**Density distribution of time interval of readmission within 30 days following discharge among patients with acute heart failure**


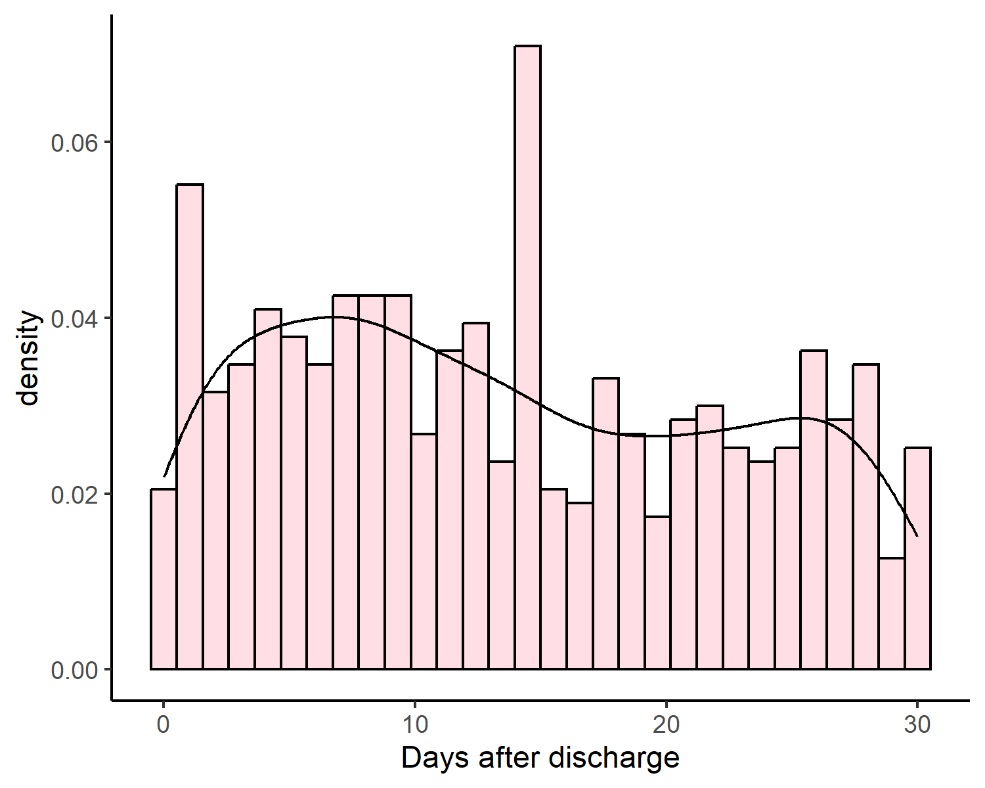


Note: days after discharge is the time interval between discharge and readmission.

**Supplementary Fig. 2.**

**Distribution of readmission causes by time interval of readmission within 30 days following discharge among patients with acute heart failure**


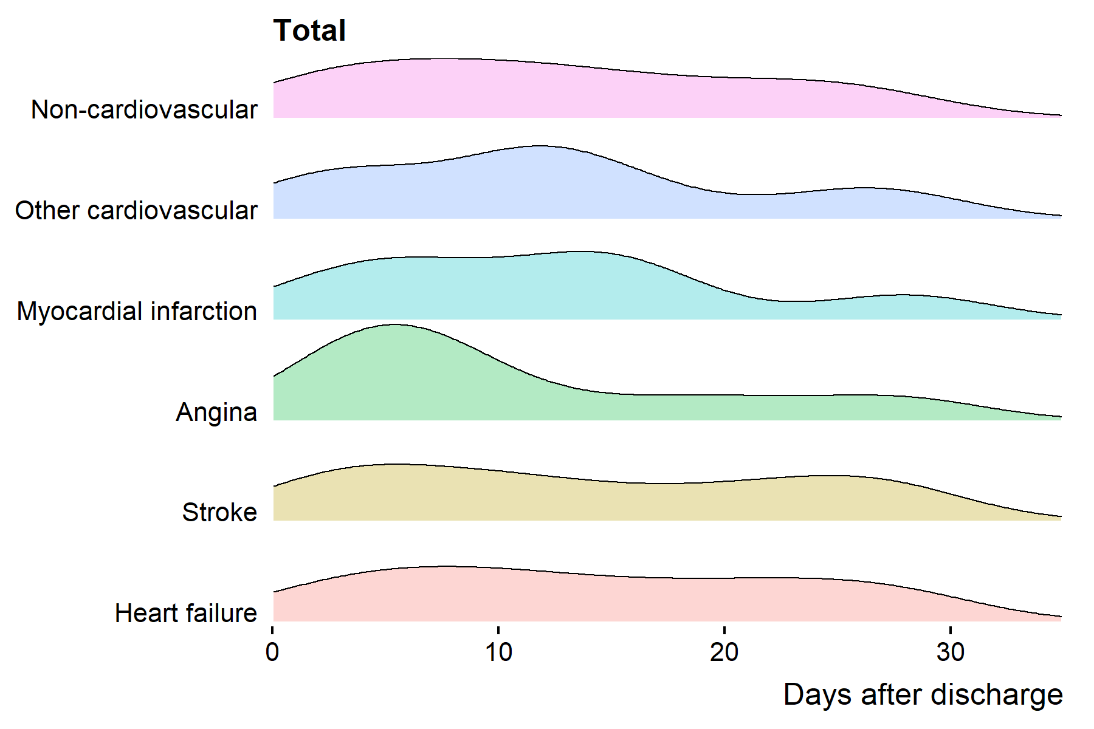


**
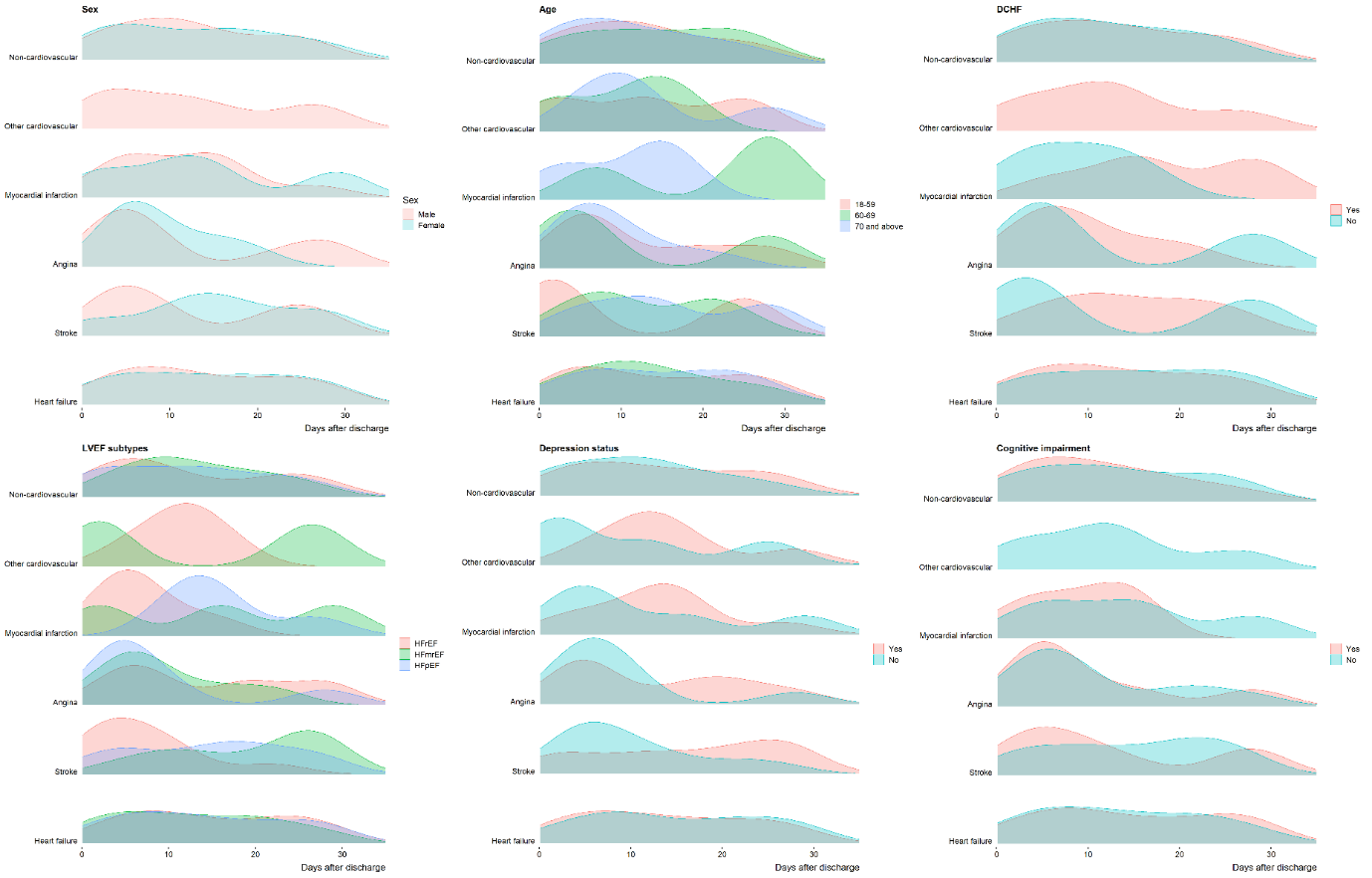
Supplementary Fig. 2A. Total**

**Supplementary Fig. 2B. Subgroups**

(A) Distribution of causes for readmission by time interval of readmission within 30 days following discharge among all study participants. (B) Distribution of causes for readmission by time interval of readmission within 30 days following discharge in subgroups by selected demographics (age, sex, DCHF) and clinical characteristics (LVEF subtypes, depression status, cognitive impairment).

Note: days after discharge is the time interval between discharge and readmission. Abbreviations: DCHF: decompensated chronic heart failure; LVEF: left ventricular ejection fraction; HFrEF: heart failure with reduced ejection fraction; HFmrEF: heart failure with mildly reduced ejection fraction; HFpEF; heart failure with preserved ejection fraction.

**Supplementary Fig. 3.**

**Distribution of time interval for heart failure (HF) readmission and non-HF readmission within 30 days following discharge among patients with acute heart failure**

**
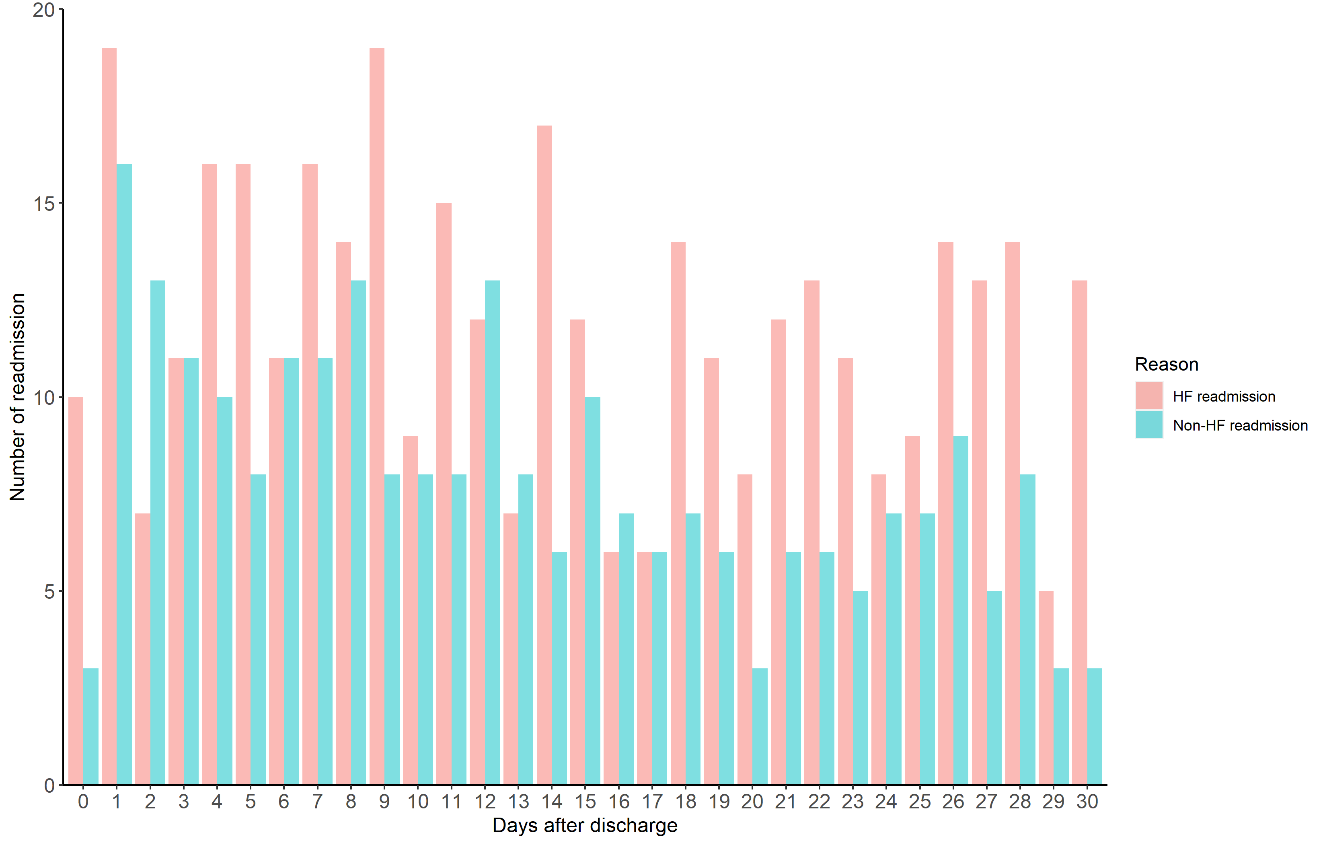
**

Note: days after discharge is the time interval between discharge and readmission.

Abbreviation: HF: heart failure.

**Supplementary Fig. 4.**

**Associated factors of 30-day readmission following discharge among patients with acute heart failure considering the competing risk**


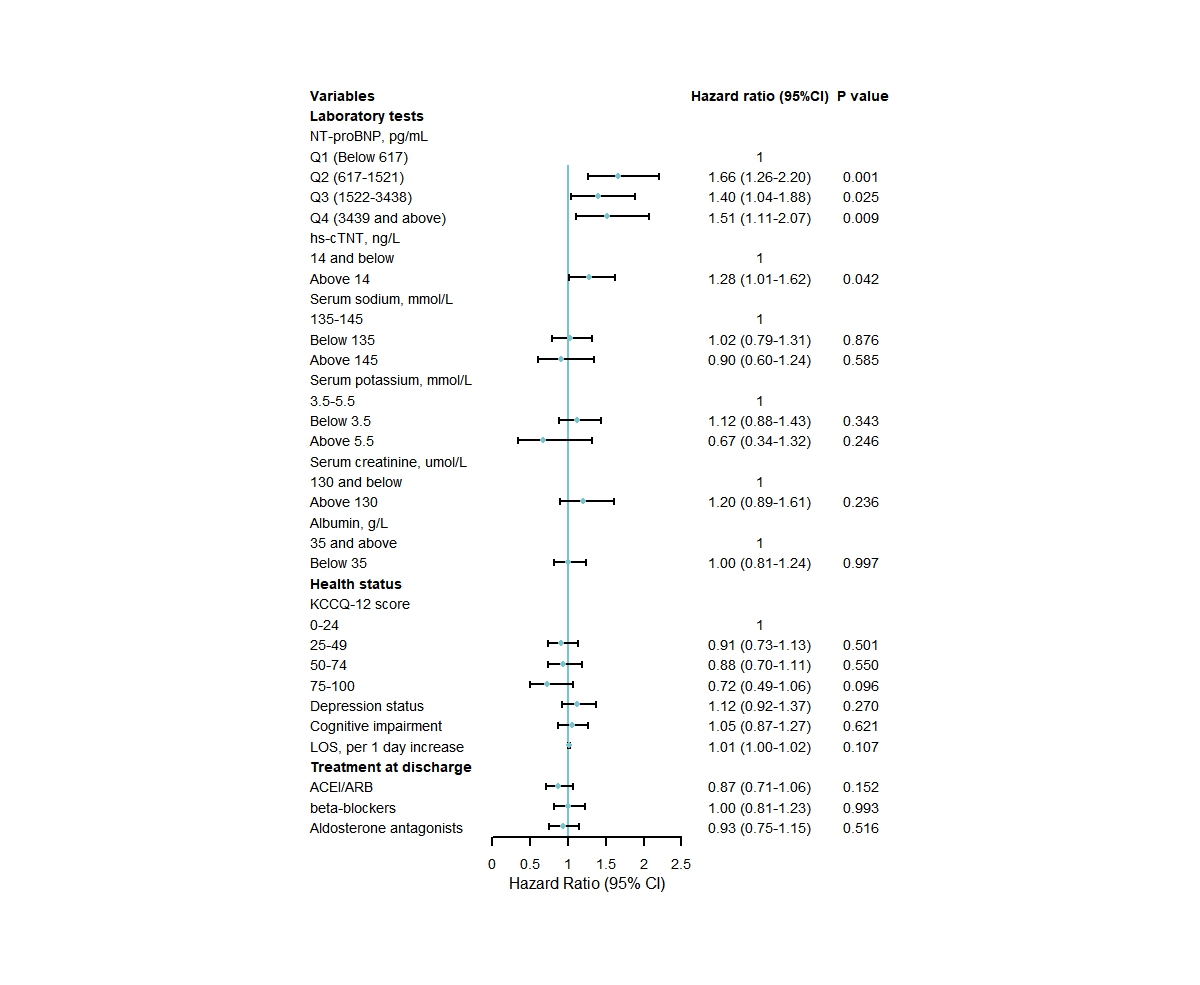

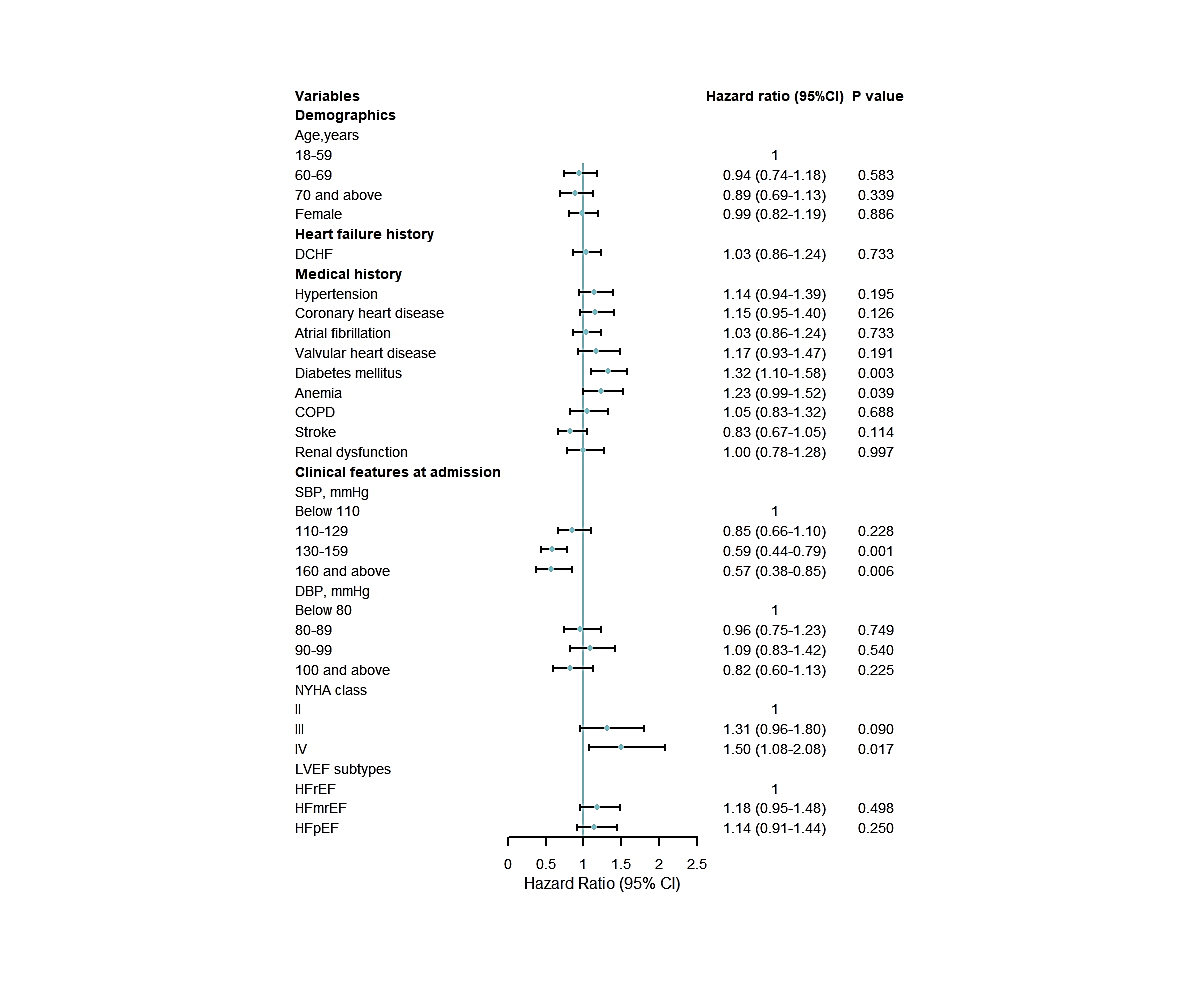


Abbreviations: CI: confidence interval; DCHF: decompensated chronic heart failure; SBP: systolic blood pressure; DBP: diastolic blood pressure; NYHA: New York Heart Association; COPD: chronic obstructive pulmonary disease; LVEF: left ventricular ejection fraction; HFrEF: heart failure with reduced ejection fraction; HFmrEF: heart failure with mildly reduced ejection fraction; HFpEF; heart failure with preserved ejection fraction; hs-cTNT: high-sensitivity cardiac troponin T; NT-proBNP: N-terminal pro-B type natriuretic peptide; KCCQ-12: Kansas City Cardiomyopathy Questionnaire-12; LOS: length of stay; ACEI: angiotensin-converting enzyme inhibitor; ARB: angiotensin receptor blocker.
